# Management of vesicovaginal fistulas after gynecologic surgery

**DOI:** 10.4274/tjod.46656

**Published:** 2017-03-15

**Authors:** Burak Tatar, Taylan Oksay, Fatma Selcen Cebe, Sedat Soyupek, Evrim Erdemoğlu

**Affiliations:** 1 Süleyman Demirel University Faculty of Medicine, Department of Gynecologic Oncology, Isparta, Turkey; 2 Süleyman Demirel University Faculty of Medicine, Department of Urology, Isparta, Turkey; 3 Süleyman Demirel University Faculty of Medicine, Department of Obstetrics and Gynecology, Isparta, Turkey

**Keywords:** Vesicovaginal fistula, gynecologic surgery, transvaginal fistula repair, abdominal fistula repair

## Abstract

**Objective:**

In developed nations, surgery, especially gynecologic procedures, is the major cause of vesicovaginal fistulas (VVFs). We retrospectively evaluated our treatment modalities for VVF repair caused by a gynecologic surgery, and discussed the reasons of selecting certain surgical techniques and their outcomes.

**Materials and Methods:**

We compared the surgical procedure preferences of surgeons and their results with patient and surgeon characteristics for the management of VVFs after an inciting gynecologic surgery in Süleyman Demirel University Hospital, Isparta over a 10-year period. The surgical procedures were undertaken in departments of urology and obstetrics and gynecology.

**Results:**

Abdominal repair was chosen for 65%, vaginal repair for 25%, and laparoscopic repair for 10% of patients. For the 75% of the patients that urologists operated, they chose the abdominal route. The mean parity number of patients who underwent abdominal repair was lower than that for vaginal repairs (p<0.05). For the patients managed with the vaginal route, 20% had a Martius flap, and 80% had a simple excision and repair. For patients operated via the abdominal route, 18% needed omental flap; no tissue interposition was used for the rest. The mean hospitalization time was less in patients managed with transvaginal repair (3.4 days) compared with transabdominal repair (9.2 days) (p<0.05).

**Conclusion:**

The choice of repair method depends on surgeon’s training (gynecology vs. urology). The vaginal route should be the first choice because it does not compromise the success rate and the mean hospitalization time is less. For the transvaginal approach, access to the lesion is the most important factor for the success of the procedure. No flap is needed for tissues that appear well vascularized.

## INTRODUCTION

Genitourinary fistulas represent significant morbidity, especially in developing nations where obstetric trauma is the major etiologic cause. The true incidence is unknown, and lack of seeking care is a major contribution for this uncertainty^(1)^. Vesicovaginal fistulas (VVFs) are the most commonly acquired fistulae of the urinary tract. In developed nations, surgery, especially gynecologic procedures, is the major cause^(2,3,4)^. The most common symptom in patients with VVF is constant urine leakage from the vagina. Predisposing factors such as chronic illnesses, previous surgery, chemotherapy, infections for postoperative fistulae are blamed, but the majority occur without any of these factors^(5)^.

Diagnosis of genitourinary fistula requires a thorough medical history and careful physical examination. Timing for presentation of symptoms may differ due to the cause and location of the fistula. Most present with leakage of urine from the vagina, immediately following injury. However, fistulae resulting from hysterectomy or cesarean delivery often present later than one or two weeks from the inciting surgery. Radiation-induced fistulas generally occur years after treatment.

**Figure 1:**
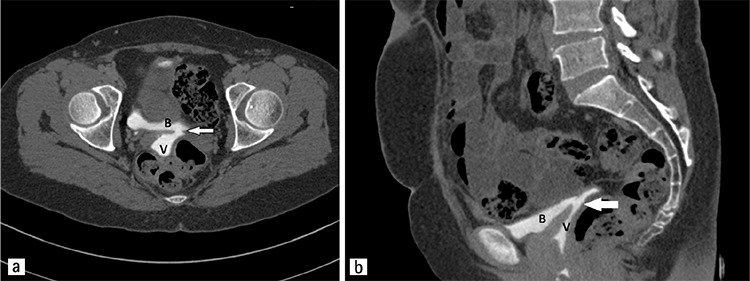
Computerized tomography urogram of a patient who underwent laparoscopic fistula repair in the delayed phase. Arrows indicate the fistula tract a) Axial image b) Sagittal image

The diagnosis can be established based on symptoms and physical examination alone (methylene blue is frequently utilized) or using imaging techniques such as cystoscopy, magnetic resonance imaging, computerized tomography (Figure 1) or ultrasound. Cystoscopy may clarify the exact anatomic origin and it is used frequently.

The type of surgical technique chosen (transvesical, transvaginal, laparoscopic or robotic), depends on surgeon experience, whether the fistula is simple or complex, and patient characteristics. Complex or high fistulas are better treated abdominally with meticulous dissection, and simple ones can be treated easily vaginally by simple excision of the devascularized tissue and multi-layer approximation of healthy tissues. Vaginal operations can be performed according to the Latzko technique as denuding vaginal epithelium and tension free re-suturing, without excision of the entire fistula tract.

In this study, we retrospectively evaluated our treatment modalities for primary VVF repair after a gynecologic surgery, and discussed the feasibility and outcomes of the surgical techniques used in our institution over a 10-year period. The aim of this single-center study was to contribute evidence to the Turkish literature by describing the surgical management of VVF treatment in one university hospital in Turkey. This may aid physicians in the selection of appropriate surgery for their patients.

## MATERIALS AND METHODS

### Patient selection

Between 2006 and 2015, a total of 20 patients were admitted to Süleyman Demirel University Hospital, Isparta, for VVF management after an inciting gynecologic surgery. Patient characteristics are outlined in Table 1. The Süleyman Demirel University Ethics Committee and Review Board approved the study (approval number: 01.06.2016/02). The study was performed in accordance with the ethical standards described in an appropriate version of the 1975 Declaration of Helsinki, as revised in 2000.

## METHODS

### Surgical techniques

### Transvaginal repair

After positioning the patient in low-lithotomy, we usually start with cystoscopy, especially if there is an uncertainty about involvement of the ureters. A Foley catheter is routinely placed to mark the fistula tract. A cystoscopic identification of the tract is made and ureteral catheterization is performed if the ureteric orifices show close proximity to the fistula.

For transvaginal repair, we prefer excision of the fistula tract and multi-layer closure with or without a Martius flap or fat pad. The excision is accomplished by taking margins with healthy tissue, approximately 1-1.5 cm in diameter. After excising the fistulous tract, the first layer sutured is the bladder mucosa, followed by the detrusor and/or prevesical or endopelvic fascia, and the third or fourth layer incorporates vaginal epithelium. Before the repair of the vaginal epithelium, a leak test is performed using diluted methylene blue. The vaginal epithelium is sutured in a perpendicular fashion with 2-0 Vicryl. For inner layers, 3-0 Vicryl is preferred. A Martius flap is used especially for larger or devascularized tissues managed with the vaginal route. The Martius flap procedure involves the use of a 2-cm wide fat pad dissected along the labium majus and tunneled as a vascular barrier under the vagina in the location of the excised fistula.

### Transabdominal repair

For the abdominal approach, after a Pfannenstiel or midline incision and exploration of the pelvis, a cystotomy is made on the dome of the bladder, and if the fistula opening is located near the ureteral orifices, ureteral stents are placed. The fistulous tract is marked by placing a 14-F Foley catheter. The fistulous tract is excised with the catheter inside. An omental or peritoneal flap is attached between the vaginal wall and bladder if necessary. The vagina is closed with 2-0 Vicryl, and the bladder is closed in 2 layers using 3-0 Vicryl.

### Laparoscopic repair

Laparoscopic surgery is performed in our institution using one umbilical port for the camera, one suprapubic 10-mm port, and two 5-mm ports bilaterally located medial to the anterior superior iliac spines. We use a 30-degree angled camera for the repair. The vesicovaginal plane is dissected until reaching the fistula tract without making a prior cystotomy. The tract is totally excised and the vagina and bladder are separately sutured using 3-0 Vicryl. A leak test is performed using diluted methylene blue.

### Urinary diversion

Urinary diversion is achieved as a part of the pelvic exenteration procedure. The ileal conduit technique is chosen for these patients. A 15-cm ileal segment is isolated using GIA staplers, and a side-to-side anastomosis is performed for the remaining bowel segments using GIA staplers. The left ureter is passed under a tunnel created in the mesentery of the sigmoid colon. A Wallace type 1 anastomosis is performed, as conjoining the distal ends of the ureters together we perform the anastomosis to the proximal end of the ileal segment. Using a Foley catheter to drain the conduit, feeding tubes are passed into each ureter and secured to the distal end of the ileal loop, and a Foley catheter is used to drain the conduit. The stoma is matured to the right side of the patient between the umbilicus and the superior anterior iliac spine.

### Statistical Analysis

Statistical analysis of the data was performed using SPSS 15.0 (SPSS Inc. Chicago, IL, USA) and p<0.05 was determined as significant. Non-parametric data were compared using Mann-Whitney U test and Pearson’s chi-square test.

The effect of age on the preference of the surgical route was calculated using the Mann-Whitney U test.

## RESULTS

Abdominal repair was chosen for 11 (55%) patients, vaginal repair for 5 (25%), laparoscopic repair for 2 (10%), and 2 patients underwent ileal conduit urinary diversion (10%). Patient characteristics for each repair type are shown in Table 2.

Of the surgeries performed by urologists, 75% were via the abdominal route and 8.3% were vaginal. Laparoscopic repairs were only performed by urologists. All patients treated by gynecologists were operated using the transvaginal route. It is clear that urologists preferred the abdominal or laparoscopic route, whereas gynecologists preferred the vaginal route, and this difference was statistically significant (p<0.05). Eighty percent of operations performed both by gynecologists and urologists were performed abdominally.

The most common single symptom was urinary incontinence (80%), followed by constant leakage of urine through the vagina (20%). Cystoscopy was performed for 85% of the patients for confirmation of the diagnosis and to evaluate the exact location of the fistula, and physical examination only sufficed for 15% of the patients.

For patients managed through the vaginal route, 20% were treated with a Martius flap, and 80% with a simple excision and repair. For patients operated via the abdominal route, 18% needed an omental flap; no tissue interposition was used for the remainder. Ureteral catheterization was performed for 5 patients, all of whom were managed via the transabdominal route. Their fistulas had proximity to ureteral orifices, 3 needed bilateral catheterization, and two needed unilateral catheterization (Table 2).

Two VVFs with obstetric etiologies were managed using abdominal excision and repair. The first patient was nulliparous and she had preterm labor at 35 weeks. The second patient had 3 prior vaginal births and had an obstructed labor due to macrosomia. A cesarean section was performed for both obstructed labors and fistulas developed thereafter.

For the patients with malignancies, both had prior history of radiotherapy. The first patient had recurrent endometrial cancer and the other had a cervical cancer and had undergone primary radiotherapy. Both had central recurrence with vesicovaginal fistulas with no evidence of extra-pelvic metastasis. Ileal conduits were performed for both patients as part of a total pelvic exenteration procedure. An infra-levator pelvic exenteration was performed for the patient with cervical cancer, whereas a supralevator procedure sufficed for the patient with endometrial cancer.

Excluding the patients with malignancies who underwent ileal conduit procedures, the mean hospitalization time was less in patients managed with transvaginal repair group (3.4 days) compared with transabdominal repair (7.9 days), and the difference was statistically significant (p<0.05). We expected a tendency of more older patients to have undergone surgery through the vaginal route, but we found no difference between the groups, even when we classified the groups by age as 25-45 years, 46-55 years, and >55 years (p>0.05). The mean parity number of the patients who underwent abdominal repair was 1.9, for vaginal repairs 3.8, and for laparoscopic repairs 2 (p<0.05).

One case was started vaginally and converted to laparotomy, after excision of the fistulous tract. For this patient, the multi-layer closure of tissues was impossible through the vaginal route due to the high location of the fistula. The patient had a recent history of a concomitant abdominal hysterectomy for myoma uteri and Burch colposuspension procedure for urinary incontinence.

One patient developed stress urinary incontinence after the repair. The patient was initially managed via the abdominal route. She was offered various treatment modalities including sub-urethral slings and bulking agents, but she refused treatment.

The only recurrence was noted in a patient who had undergone laparoscopic surgery. A transabdominal repair was successfully performed 4 weeks after the first surgery. No flap was used due to the well-vascularized appearance of the tissues. The etiology of the fistula was abdominal hysterectomy for myoma uteri, which performed 6 weeks earlier than the first repair attempt. No recurrence occurred during her 1-year follow-up.

## DISCUSSION

In this retrospective study, we evaluated only the surgical approach for the management of VVFs; therefore, patients who were conservatively treated were out of the scope of this study. There are controversies as to whether the treatment should be conservative or surgical. In the minority of cases, the fistula may close spontaneously after 2-4 weeks of urethral catheterization, especially if the fistula is detected early (no epithelization on the fistula tract) and the diameter is small^(6)^. Timing of the repair is also important. When identified before 72 hours after iatrogenic cystotomy, VVF can be repaired immediately. If the diagnosis of a small fistula is established late and the fistula is epithelized, electrocoagulation of the mucosal layer and catheterization may lead to closure in up to 75% of cases^(7)^.

In a recent report from Turkey, outcomes of 53 cases with VVFs were discussed and none of the fistula closed with conservative management^(8)^. The use of fibrin sealants for closure of small fistulae has also been reported^(9,10)^. Fibrin glue has also been successfully used instead of Martius flap in cases when tissue interposition was needed^(11)^. However, in our clinic, we do not have such experience. As reported in the study of 52 cases by Kapoor et al.^(12)^, the mean blood loss and postoperative pain may be less, and the mean hospital stay may be shorter for transvaginal repair compared with transabdominal repair, especially in non-complicated cases.

For vaginal approach, our clinic prefers simple excision and repair, and the long-term success of this approach seems excellent, because none of the fistula recurred. In the literature, the success of transvaginal repair ranges from 70% to 100%^(13,14,15)^. A large prospective cohort study from Africa that compared 1273 abdominal and vaginal genitourinary fistula repairs found that vaginal route repairs were associated with increased risk of failure in closing the fistula compared with the abdominal route. However, the follow-up was 84 to 99 days, nearly 20% of the patients had a degree of genital mutilation; there was extensive scarring in 7.7% of patients operated via vaginal route versus 3.5% of patients operated via the abdominal route, only 3.69% of the patients underwent abdominal surgery, and finally the population comprised VVFs and all types of genitourinary fistulas^(16)^.

Martius flap is used only for 20% of patients, and is chosen for tissues that appear as devascularized. The success of this technique seems more than 90%^(2)^. The vaginal approach may also be possible for supra-trigonal fistulas, depending on the experience of the surgeon^(17)^.

The abdominal route should be considered for larger, more complex or recurrent fistulas. Large fistulas (>2 cm) and those close to ureteric orifices may be considered as “complicated” or “complex” and there is no consensus as to which fistulas are considered as complicated. The success abdominal repair ranges between 90-100%^(14)^. Despite the proven long-term results of the vaginal approach for VVFs, there is a tendency in our clinic to perform abdominal repairs, especially for cases that urologists perform. However, that difference may be according to a selection bias; urologists generally deal with more complicated cases. Both patients who received prior radiotherapy underwent surgery with urologists and gynecologists together, and the laparoscopic failure of closure was performed by the urologists. The mean number of births was higher in transvaginal repair group compared with the transabdominal group, and this may be one of the factors for surgeons to consider when choosing either route.

The laparoscopic approach, as an alternative, results in less morbidity and recovery is faster than the transabdominal route. The success of this approach is comparable with open procedures^(18)^. However, it requires advanced skills such as suturing in non-ergonomic angles.

There are no randomized controlled studies to evaluate whether abdominal, laparoscopic or vaginal approach is superior. We found that the mean hospitalization time was less for vaginal repairs, and avoiding a laparotomy may also reduce the rate of complications, although we did not encounter any. It would be appropriate to repeat classic teaching that if the fistula is large, complex, ureteral involvement is suspected, an abdominal approach may be preferred over a vaginal approach.

No de-novo stress urinary incontinence was reported in the vaginal or laparoscopically managed groups, but there was one in the trans-abdominally managed group. After excluding patients with urinary diversion, the rate was 11%. After fistula surgery, most residual incontinence is thought to be stress urinary incontinence^(19)^. Nevertheless, there are some data in favor of detrusor instability as a major contributing factor. A report from the United Kingdom mentioned a post-repair stress urinary incontinence rate of about 11%, whereas detrusor instability was documented as 50% in this population^(15)^. In another report, it was indicated that both stress and urge symptoms occurred in similar numbers in patients after a repaired fistula^(20)^. A report from Australia indicated a 23.9% rate of urinary incontinence after repair. In developing countries where obstetric fistulas are major contributors, this rate seems much higher^(21,22)^. A series of 318 consecutive patients from Addis Ababa, where the main inciting factor was obstetric trauma for the fistula, reported an immediate post-operative incontinence rate of 33%^(14)^. Bladder neck involvement and proximal urethral contribution to fistula can be considered as risk factors for post-closure incontinence^(23)^.

In the current study, abdominal hysterectomies alone contributed to 65% of the fistulas and hysterectomy with Burch colposuspensions caused 10% of the fistulas. These rates are closer to the rates of developed nations as gynecologic surgeries, mainly abdominal hysterectomies, rather than obstetric traumas, are causes of the fistulas^(4,12,24)^. The effect of colposuspension as a contributing factor could not be analyzed because of concomitant hysterectomies. It is crucial to meticulously dissect the bladder from the cervix and proximal vagina, suturing only vagina without incorporating the detrusor fibers and avoiding excessive use of electrocautery while working in close proximity to the bladder, because usually no cystostomy or urinary tract injury is encountered during hysterectomies causing fistulas.

The incidence of fistulas caused by radiotherapy for malignant conditions such as cervical cancer and endometrial cancer is about 5%^(25)^. For these circumstances, urinary diversion may be chosen. Simple repair is generally not suitable for these patients because of fibrosis, unhealthy tissue and distorted anatomy may not be amenable to re-approximation. If a repair is to be attempted, an intervening well-vascularized flap is strongly recommended. Successful fistula repair is reported as between 70 and 100% in non-irradiated patients, and between 40% and 100% for patients who had prior radiotherapy^(14)^. Urinary diversions are much preferred for patients with cancer who have previously been irradiated. Both ileal conduits performed at our institution were as part of pelvic exenteration procedures for central recurrence of tumors.

**Study Limitations**

One of the major limitations of the study is that the number of the patients was small for each group to be compared; for example, there were only two cases managed laparoscopically among twenty patients. Also, the retrospective design of our study may be a drawback, but a prospective trial for management of vesicovaginal fistulas is hard due to its rarity. In our opinion, a multi-center trial design is more appropriate for prospective trials related with this problem.

## CONCLUSION

Nearly all VVFs in this series resolved with primary surgery, regardless of the approach. No flap is needed for tissues that appear well vascularized. The mean hospitalization time is less in patients managed with transvaginal repair compared with transabdominal repair, and this difference emphasizes the vaginal route as the first choice without compromising the success rate.

## Figures and Tables

**Table 1:**
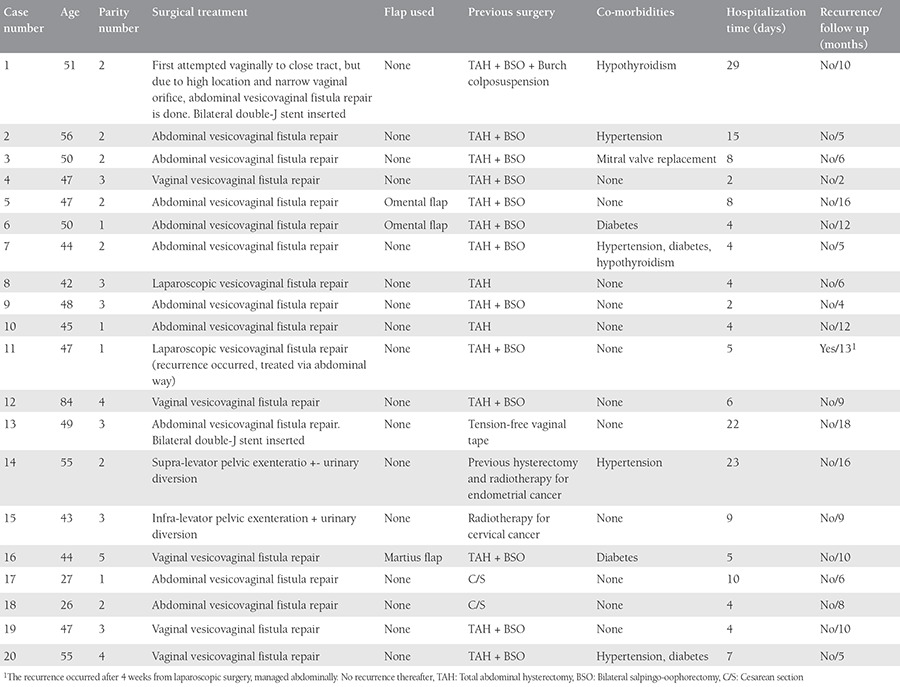
Patients’ characteristics

**Table 2:**
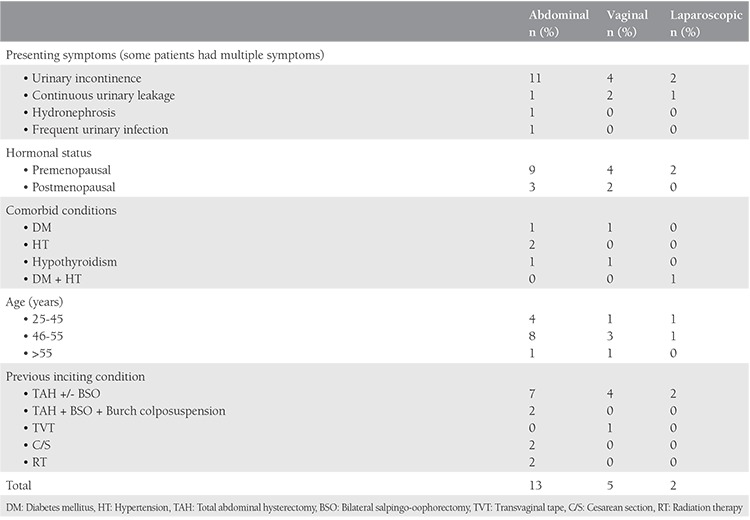
Vesicovaginal fistula characteristics stratified by repair type
